# 5-CQA and Mangiferin, Two Leaf Biomarkers of Adaptation to Full Sun or Shade Conditions in *Coffea arabica* L.

**DOI:** 10.3390/metabo10100383

**Published:** 2020-09-26

**Authors:** Teerarat Duangsodsri, Luc Villain, Ialy Rojo Vestalys, Serge Michalet, Cécile Abdallah, Jean-Christophe Breitler, Mélanie Bordeaux, Andres Mauricio Villegas, Marson Raherimandimby, Laurent Legendre, Hervé Etienne, Benoît Bertrand, Claudine Campa

**Affiliations:** 1 IRD, CIRAD, Univ. Montpellier, IPME, F-34394 Montpellier, France; teerarat.duangsodsri@ird.fr (T.D.); ialyvestalys@gmail.com (I.R.V.); cecile.abdallah@ird.fr (C.A.); 2 IPME, Univ. Montpellier, IRD, CIRAD, F-34394 Montpellier, France; luc.villain@cirad.fr (L.V.); jean-christophe.breitler@cirad.fr (J.-C.B.); herve.etienne@cirad.fr (H.E.); benoit.bertrand@cirad.fr (B.B.); 3 CIRAD, UMR IPME, F-34398 Montpellier, France; 4 Faculté des Sciences, Université d’Antananarivo, BP-566, Antananarivo 101, Madagascar; marson.rahery@yahoo.com; 5 CNRS UMR 5557, Univ. Lyon 1 & INRA UMR 1418, Université de Lyon, F-69622 Villeurbanne, France; serge.michalet@univ-lyon1.fr (S.M.); laurent.legendre@univ-lyon1.fr (L.L.); 6 CIRAD, INECOL, Clúster BioMimic, Xalapa 91073, Veracruz, Mexico; 7 Fondation NicaFrance, Managua, Nicaragua; research@fundacionnicafrance.org; 8 Calle 15, 9-18 Conjunto Mirador de las Lomas, Villamaría 175008, Caldas, Colombia; andresvillegas75@gmail.com

**Keywords:** adaptive response, biomarkers, *Coffea arabica* L., full-sun conditions, light response, phenolic compounds

## Abstract

Phenolic compounds are involved in plant response to environmental conditions and are highly present in leaves of *Coffea arabica* L., originally an understory shrub. To increase knowledge of *C. arabica* leaf phenolic compounds and their patterns in adaptation to light intensity, mature leaves of Ethiopian wild accessions, American pure lines and their relative F1 hybrids were sampled in full sun or under 50% shade field plots in Mexico and at two contrasting elevations in Nicaragua and Colombia. Twenty-one phenolic compounds were identified by LC-DAD-MS^2^ and sixteen were quantified by HPLC-DAD. Four of them appeared to be involved in *C. arabica* response to light intensity. They were consistently more accumulated in full sun, presenting a stable ratio of leaf content in the sun vs. shade for all the studied genotypes: 1.6 for 5-CQA, F-dihex and mangiferin and 2.8 for rutin. Moreover, 5-CQA and mangiferin contents, in full sun and shade, allowed for differentiating the two genetic groups of Ethiopian wild accessions (higher contents) vs. cultivated American pure lines. They appear, therefore, to be potential biomarkers of adaptation of *C. arabica* to light intensity for breeding programs. We hypothesize that low 5-CQA and mangiferin leaf contents should be searched for adaptation to full-sun cropping systems and high contents used for agroforestry systems.

## 1. Introduction

Arabica coffee (*Coffea arabica* L.) is a cash crop of major social–economic importance throughout the intertropical zone. Wild *C. arabica* is an understory bush that originated in the southwest Ethiopian highlands and in the Boma Plateau forests in southern Sudan [1]. However, from its early domestication in Yemen (secondary dispersal center [2]) to the most recent cultivation of homozygous varieties, such as the American pure lines, *C. arabica* has been mainly bred for full-sun growing conditions, particularly in intensive cropping systems. It has recently been shown that wild *C. arabica* germplasm resulting from a recent speciation process has low genetic diversity [3]. Cultivated material has particularly low genetic diversity because traditional cultivars derive from only two narrow genetic groups that spread from Yemen: var. Typica and var. Bourbon [4]. From the 1970s on, coffee breeding programs aimed to introgress coffee leaf rust (*Hemileia vastatrix*) resistance genes from accessions of the hybrid of Timor—a natural interspecific hybrid of *C. arabica* and *C. canephora* [5]—leading to the creation of the Catimor and Sarchimor cultivar families [6]. Nevertheless, the genetic base of *C. arabica* cultivars remains narrow since very few traditional Timor hybrid progenitors or traditional recurrent parents were used in these introgression breeding programs [7].

A recent study sequenced and independently assembled the two-component genome of the allotetraploid *C. arabica* species (putatively deriving from two other species, *C. canephora* and *C. eugenioides*). Genome-wide SNP genotyping of the genetic diversity was also performed in cultivated coffee germplasm and in wild populations [3]. The study revealed a weak population structure due to low frequency-derived alleles and a cline of genetic diversity reflecting west-to-east geographical distribution, from the center of origin in East Africa to the Arabian Peninsula. Two main genetic groups were identified: the eastern and wilder plants, consisting in two populations, “Sheka” and “Jimma Bonga”, and the “Harar-Yemen” group corresponding to the very early domestication process of *C. arabica*. The eastern group thus appeared to be a valuable untapped genetic reservoir for the improvement of cultivated *C. arabica* varieties. It could, for example, be valued in breeding programs such as those started in the 1990s that led to most current improved F1 hybrids with the best yields and beverage quality obtained using controlled crosses between wild Ethiopian progenitors and American cultivars [6].

Current research in many coffee growing countries aims to improve, or reintroduce, agroforestry cropping systems because of the many ecosystem services they deliver [8] and their resilience in the face of climate change in the most exposed regions, mainly at medium to low elevations [9]. However, for a variety of reasons, the agroforestry model, which may limit profitability in countries such as Brazil or food plant intercropping in some African countries, is not always applicable. To develop unshaded coffee cropping systems, new *C. arabica* varieties must be developed to improve sustainability and enable the agro-ecological transition by introducing genotypes that are naturally resistant to abiotic and biotic stresses. To achieve this aim, new high-throughput phenotyping tools are needed to identify candidate progenitor parents among wild and cultivated germplasms.

Secondary metabolites (SMs) are among the main tools that enable plants to adapt to biotic and abiotic stresses [10]. Their accumulation is finely tuned to perceived environmental cues [11]. They belong to different chemical classes, including phenolic compounds, alkaloids and terpenes. A large body of literature is devoted to phenolic compounds as they are involved in plant resistance and acclimation to environmental constraints. Different mechanisms are involved, including strong antioxidant activity that enables them to quench the reactive oxygen species (ROS) emitted during any form of stress, their capacity to absorb UV [12,13,14] and their toxicity to plant pests [15]. They are key players in the adaptation of *Arabidopsis thaliana* leaves to intense light [16]. Caffeoylquinic acids (CQAs) and chlorogenic acids (CGAs) described as lignin precursors and carbon reservoirs [17], accumulate in greater quantities in response to temperature and light in *Solanum tuberosum* leaves and in *Capsicum annuum* seedlings [18,19].

In coffee plants, SMs are well described in the economically valuable beans because of their contribution to coffee beverage quality and human health [20,21,22]. They include a variety of CGAs (esters of hydroxycinnamic acids—mainly 5-CQA), alkaloids such as caffeine and its derivatives [23,24], lipids [25,26] and volatile organic compounds [27]. Studies on SMs in leaves are rare and are often limited to caffeine and CGAs [28,29]. Recently, the presence of other phenolics, such as flavonoids [30] and the xanthone mangiferin [31,32], have been reported, promoting the use of coffee leaves like tea leaves, and leading to studies on leaf nutraceutical compounds [33,34]. However, studies on the role of leaf phenolic compounds in coffee adaptation to environmental constraints are rare, with the exception of those on CGAs in the response to cold of tolerant genotypes [35] and on glycosylated flavonoids in high light tolerance of Catuai, an American pure line variety [30].

The aim of the present study was thus to increase current knowledge of *C. arabica* leaf phenolic compounds and their patterns in plant adaptation to light intensity.

## 2. Results

### 2.1. Identification of Phenolic Compounds in Mature Leaves of C. arabica Using LC-MS^2^

To enable the most thorough analysis possible of the phenolic compounds present in the mature leaves of *C. arabica*, even those accumulated at very low concentrations, a LC-DAD-MS^2^ analysis was conducted on leaf extracts from a *C. arabica* variety (cv. Marsellesa) grown under full sun. Only 23 substances reached the automatic pseudomolecular ion detection threshold that triggers introduction of ionized substances in the collision cell for fragmentation pattern acquisition. All generated a fragmentation pattern after positive ionization, while only 21 yielded a fragmentation pattern under negative ionization (Table 1). The identity of 10 compounds was confirmed by parallel analysis of pure standards, while basic structural information is suggested for the others based on their high-resolution mass, fragmentation pattern and UV spectrum.

Compounds **2**, **5** and **6** were characterized in negative mode by a pseudomolecular ion [M − H]^−^ at *m*/*z* 353.0901 and in positive mode ([M+H]^+^) *m*/*z* 355.1002, corresponding to a molecular formula of C_16_H_18_O_10_. The three compounds share the same UV spectrum with a shoulder at 300 nm and a maximum at 325 nm. These characteristics correspond to mono-CQA isomers. Compound **5** was identified as 5-*O*-CQA by comparison with a standard, while compounds **2** and **6** were identified as 3-*O*- and 4-*O*-CQA, respectively, based on the literature describing their retention time (RT) elution order and their specific MS^2^ spectra in negative mode [36,37]. Using the same combined analyses, **9** and **11** were identified as 5-*O*-coumaroylquinic acid (5-CoumQA) and 5-*O*-feruloylquinic acid (5-FQA), respectively. Compound **3** was characterized by *m*/*z* 409.1107 and 407.1012 in positive and negative mode, respectively, suggesting a molecular formula of C_19_H_20_O_10_. The UV absorption spectrum (λ_max_ at 296 nm) and complex MS^2^ fragmentation suggest that **3** is a glycosylated benzophenone-*C*-heteroside, tentatively identified as iriflophenone 3-*C*-β-glucoside [38]. By comparison with standards, peaks **4** and **8** were identified as (+)-catechin and (−)-epicatechin, respectively, and peak **10** as mangiferin (Mang). Compound **23** showed a UV spectrum similar to Mang, indicating that **23** is a Mang derivative. Its *m*/*z* under positive and negative modes corresponded to Mang + C_7_H_4_O_2_. Compared to Mang, it displayed an additional fragment ion at *m*/*z* 385 in negative ionization mode corresponding to a loss of [parahydroxybenzoate+H_2_O], and a major fragment ion at *m*/*z* 121 in positive mode, corresponding to a benzoic acid ion ([C_7_H_5_O_2_^.^]^+^). This suggests that **23** is mangiferin parahydroxybenzoate (Mang-OHbenz). Peak **12** was characterized by a pseudomolecular ion at *m*/*z* 595.1643 and at *m*/*z* 593.1555, in positive and negative mode, respectively, leading to a molecular formula of C_27_H_30_O_15_. Its UV spectrum (λ_max_ at 265 and 330 nm) resembles a di-hexosyl flavone [39]. Its MS^2^ fragmentation in positive and negative modes suggests that **12** is a flavone-di-*C*-hexose (F-dihex).

Peaks **13**, **14**, **16** and **18** shared the same UV spectra (λ_max_ at 257 and 353 nm with **13** and **14** but with an additional shoulder at 300 nm) indicative of 3-*O*-substituted flavonols [39]. Compounds **14**, **16** and **18** were identified as quercetin-3,4-di-*O*-glucoside (Q-diglu), rutin (quercetin-3-*O*-rutinoside) and quercetin-3-*β*-D-glucoside (Q-glu), respectively, by comparison with standards. Peak **13** showed the same UV spectrum as compound **14** but eluted earlier. The two substances therefore shared the same UV-absorbing moiety. In addition, **13** displayed pseudomolecular ions at *m*/*z* 773.2129 and 771.2036 in positive and negative mode, respectively (this is a gain of 146 amu compared to **14**, corresponding to the addition of a deoxyhexose). It was therefore annotated as quercetin-3-*O*-dihexose-deoxyhexose (Q-dihex-dhex).

Peaks **15** and **19** only fragmented in positive mode because of their low abundance and/or lesser stability of their deprotonated forms. They shared the same UV spectrum (λ_max_ at 265 and 346 nm) and MS^2^ fragmentation as a base peak at *m*/*z* 287, indicative of 3-*O*-substituted kaempferol derivatives. Compound **15** showed fragment ions at *m*/*z* 612 (loss of deoxyhexose), 449 (loss of deoxyhexose + hexose) and 287 (loss of deoxyhexose + 2 hexoses) and was annotated as kaempferol-3-*O*-dihexose-deoxyhexose (K-dihex-dhex). Similarly, compound **19** was annotated as kaempferol-3-*O*-hexose-deoxyhexose (K-hex-dhex).

Peak **17** showed pseudomolecular ions at *m*/*z* 579.1472 and 577.1386 (molecular formula of C_30_H_26_O_12_) and exhibited a weak intensity λ_max_ at 276 nm, characteristic of catechin derivatives. Based on its MS^2^ fragmentation, it was annotated as catechin dimer (procyanidin). Peaks **20**, **21** and **22** had similar UV spectra (λ_max_ at 324–327 nm and a shoulder at 297–300 nm) and the same molecular formula of C_25_H_24_O_12_. Compound **21** was identified as 3,5-*O*-dicaffeoylquinic acid by comparison with a standard (3,5-diCQA) and, following [40], **20** and **22** were identified as 3,4-*O*-dicaffeoylquinic acid (3,4-diCQA) and 4,5-*O*-dicaffeoylquinic acid (4,5-diCQA), respectively.

To sum up, the LC-MS^2^ analysis identified two metabolites belonging to the purine alkaloids, theobromine (**1**) and caffeine (**7**), eight being CGA derivatives (N°**2,5,6,9,11,20,21,22**), three being benzophenones and related xanthones (N°**3,10,23**) and ten being flavonoids (N°**4,8,12–19**).

### 2.2. Influence of Light Intensity on Leaf Phenolic Content in One C. arabica Cultivar at Low or High Elevation

**Figure 1:**
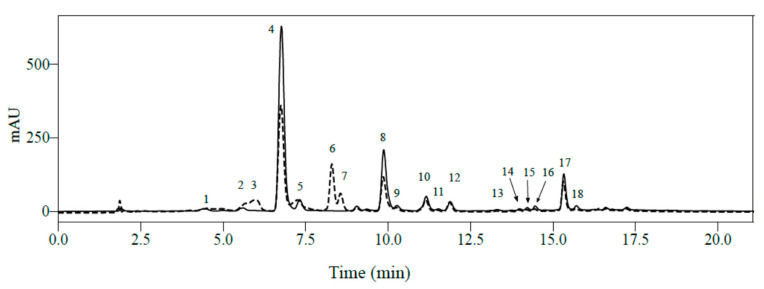
Superimposed chromatograms at 320 nm (solid line) and 280 nm (dotted line) of mature *Coffea arabica* leaves. Separation was carried out on a Agilent Eclipse XDB C18 column with methanol and 2% acetic acid in water as eluents. 1: theobromine; 2, 3-CQA; 3, (+)-catechin; 4, 5-CQA; 5, 4-CQA; 6, caffeine; 7, (-)-epicatechin; 8, mangiferin; 9, FQA; 10, flavone-di-C-hexose (F-dihex); 11, quercetin-dihexose-deoxyhexose (Q-dihex-dhex); 12, quercetin-diglucoside (Q-diglu); 13, kaempferol-dihexose-deoxyhexose (K-dihex-dhex); 14, kaempferol-hexose-deoxyhexose; 15, 3,4-diCQA; 16, 3,5-diCQA; 17, quercetin-rutinoside (rutin); 18, 4,5-diCQA.

Regardless of the elevation (650 or 1250 m asl) or light intensity (full sun or 50% shade), HPLC-DAD analyses of mature leaves from *C. arabica* cv. Marsellesa enabled quantification of 16 major phenolic compounds among the 21 identified by LC-MS^2^ in positive mode: seven CGAs, one xanthone and eight flavonoids (Figure 1). Among them, leaf concentrations of 5-CQA were the highest regardless of elevation and light conditions. Values ranged from 3.0 to 4.8 mg.100 mg^−1^ dry weight (DW) in leaves grown in full sun at high and low elevation, respectively (Table S1). Catechin, epicatechin and mangiferin had intermediate leaf contents, with the corresponding ranges (1.48–2.27), (0.79–1.52) and (1.01–1.69) mg.100 mg^−1^ DW, respectively. All other phenolic compounds were quantified at less than 1 mg.100 mg^−1^ DW regardless of elevation and light conditions.

**Figure 2:**
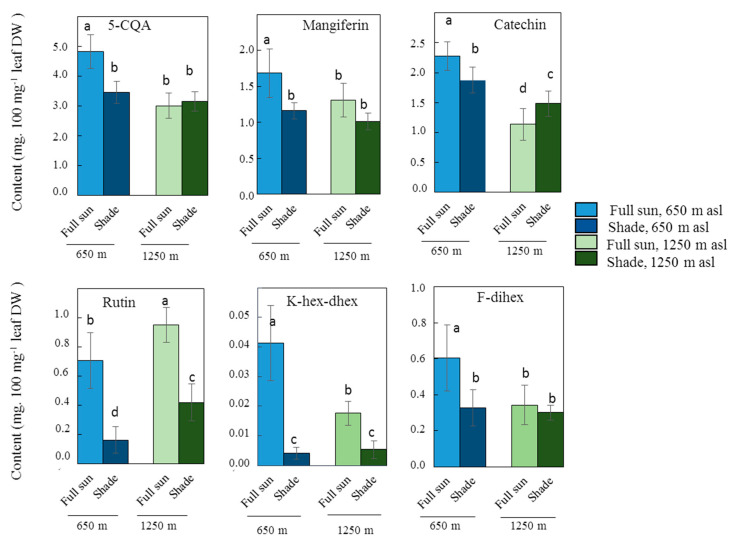
Effect of shade and elevation on the leaf content, expressed in mg.100 mg^−1^ leaf dry weight, of the more discriminant phenolic compounds in *C. arabica* cv. Marsellesa. Error bars represent ± standard deviation of mean (*n* = 10 for full-sun plants, *n* = 9 for shaded plants). Different letters indicate significant differences at *p* < 0.05 (Newman–Keuls test) among means.

Among CGAs, only 5-CQA, 3,5-diCQA and 4,5-diCQA presented significant differences in leaf content (*p* < 0.05) depending on light intensity (Table 2). Like all other CGAs, except FQA, they also presented significant differences in leaf content depending on the elevation, and significant interactions were observed between two factors, light and elevation. 5-CQA had the most discriminating CGA leaf contents by far not only for light conditions but also for elevation, suggesting that other abiotic factors linked to elevation also significantly influence 5-CQA leaf content. Indeed, a significant difference in 5-CQA content depending on light was observed at low elevation, as in full sun, concentrations were 40% higher than those in the shade (Figure 2).

With the exception of catechin, the concentration of each of the flavonoids in leaves varied significantly (*p* < 0.05) depending on the light conditions. The concentration of the eight compounds quantified also differed significantly depending on elevation, with no significant interaction between light and elevation, with the exception of the three glycosylated quercetins (Table 2).

A significant effect of light was only observed in concentrations of epicatechin, with a low *F* value of 6.0 (Table 2), and a significant difference in concentration in leaves growing in the sun and in the shade was only seen at high elevation, the concentrations being the highest in the shade (Table S1). There were always significant differences in leaf catechin content between plants grown in the sun and in the shade at both elevations, but inversed: full-sun leaf catechin content was highest at low elevation and lowest at high elevation (Table S1; Figure 2).

Of the two glycosylated kaempferols quantified, K-hex-dhex presented by far the most variable leaf contents depending on light (Table 2). The leaf contents were significantly higher in full sun than in the shade at both elevations: 10-fold higher at low elevation and 3-fold higher at high elevation (Figure 2).

Like K-hex-dhex, rutin leaf contents were largely and significantly higher in full sun than in the shade at both elevations: 4-fold higher at the low elevation and 2.3-fold higher at the high elevation (Figure 2). It should be noted that there were major significant effects of light and elevation on the rutin content in leaves, but without any significant interaction between the two abiotic factors.

At low elevation, F-dihex leaf content was 1.81-fold higher in full sun than in the shade, while no significant difference was observed at high elevation (Table S1; Figure 2). Among the glycosylated flavonoids, this compound was therefore less discriminating of light conditions than K-hex-dhex and rutin. However, the concentrations of all three glycosylated quercetins were highly variable for light conditions, rutin content being by far the most variable (Table 2).

Concentrations of mangiferin, the only xanthone derivative quantified in this study, differed significantly in leaves depending on both light conditions and elevation, but no significant interaction was found between the two (Table 2). Significant differences were observed at both elevations with higher concentrations in the sun than in the shade: 1.45-fold higher in full sun than in the shade at the low elevation, and 1.30-fold higher at the high elevation (Figure 2; Table S1).

**Figure 3:**
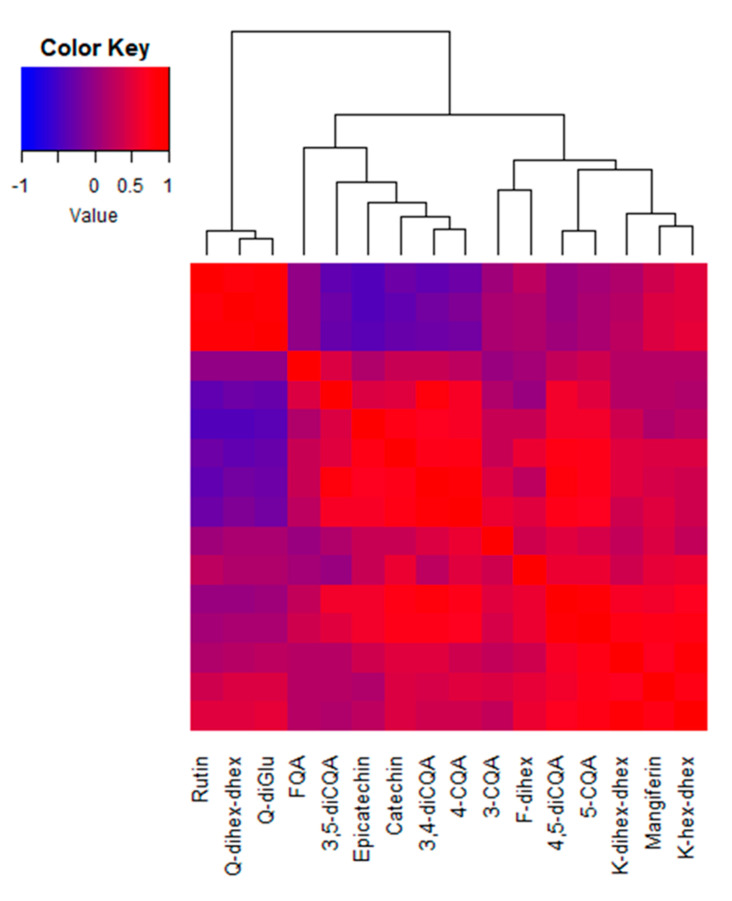
Heatmap of the Pearson’s correlation coefficients among leaf phenolic compound contents in *C. arabica* cv. Marsellesa. Samples of all elevations and light conditions at the two experimental plots in Mexico (n = 38) were used to conduct the analysis. A symmetrical heatmap was computed and a dendrogram computed by linkage clustering using the Euclidian distance measures and reordering using row (equal to column) means. Q: quercetin; FQA: feruloyl quinic acid; CQA: caffeoyl quinic acid; F: flavone; K: kaempferol; hex: hexose; dihex: dihexose; dhex: deoxyhexose.

A heatmap was drawn using the correlation coefficients among leaf contents of these 16 phenolic compounds (Figure 3). A strong correlation was noticed between the three flavonoids belonging to glycosylated quercetins: Q-dihex-dhex, Q-diGlu and rutin. These clustered away from all other phenolic compounds, including 5-CQA and the three other glycosylated flavonols. The other large cluster was subdivided into substances whose contents negatively correlated with those of the quercetin analog/rutin cluster and the substances whose contents displayed no correlation with this cluster. Within this large cluster, it can be noticed that the contents of the flavanols, epicatechin and catechin are highly correlated (*R* = 0.80; *p* < 0.05) as well as those of 5-CQA and 4,5-diCQA (*R* = 0.87) and the two glycosylated kaempferols (*R* = 0.85) that sub-clustered with mangiferin.

Together, based on ANOVA and correlation analysis of the leaf phenolic contents of the Marsellesa cultivar, we retained six major compounds with significant differences according to light conditions at one or both elevations: 5-CQA, catechin, K-hex-dhex, rutin, F-dihex and mangiferin.

### 2.3. Influence of Light Intensity on the Leaf Phenolic Content of Numerous C. arabica Genotypes Grown in Different Environments

**Figure 4:**
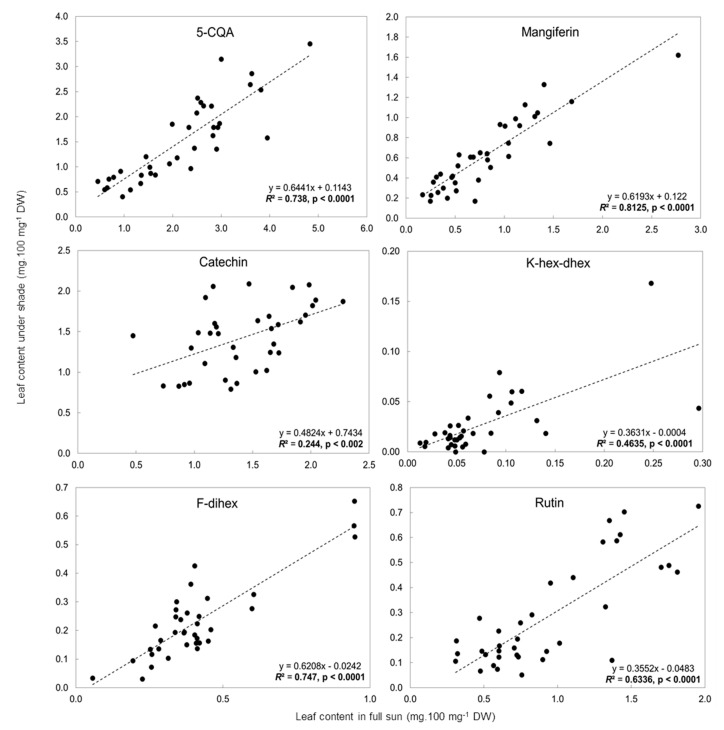
Linear regression analysis between in full-sun and under shade leaf contents (mg.100 mg^−1^ leaf DW) of 5-CQA, mangiferin, catechin, rutin, K-hex-dhex and F-dihex in Nicaragua and Colombia. The *r*^2^ values in bold indicate significant correlations (*p* < 0.05).

The same 16 phenolic compounds analyzed in the mature leaves of cv. Marsellesa in the Mexican field trials were identified in all *C. arabica* genotype samples (7 American pure lines, 8 Ethiopian wild accessions and 19 of their F1 hybrid clones) in trials in both Nicaragua and Colombia (data not shown). Correlation and linear regression analyses were conducted on the concentration (Table S2) of the six previously selected phenolic compounds discriminating full-sun and 50% shade conditions. These analyses concerned all the genotypes, those studied in the Nicaragua and Colombia trials and cv. Marsellesa grown in the two Mexican field trials (Figure 4).

Except for catechin and K-hex-dhex, leaf phenolic contents in the sun and in the shade were significantly correlated (*r*^2^ > 0.5; *p* < 0.05). The *r*^2^ of mangiferin was particularly high (*r*^2^ = 0.812), as well as that of F-dihex (*r*^2^ = 0.747) and 5-CQA (*r*^2^ = 0.738) and to a lesser extent that of rutin (*r*^2^ = 0.634). The high correlations between these four compounds indicated that, whatever the environmental conditions linked to the geographical location of the trial, there was a strong and stable relation between the leaf contents evaluated in full sun and in the shade (50% light exclusion in all the trials). However, marked variability in leaf content was observed among the genotypes. In the shade, 5-CQA and mangiferin leaf contents varied from 0.41 to 2.86 mg.100 mg^−1^ DW and 0.17 to 1.62 mg.100 mg^−1^ DW, respectively (Table S2). For 5-CQA, mangiferin and F-dihex, the slope of the regression line was similar: 0.64, 0.62 and 0.62, respectively. For rutin, the ratio of leaf content in the sun to that in the shade was much higher and the slope of the regression line was much lower: 0.36 (Figure 4).

### 2.4. Influence of C. arabica Genetic Groups on Leaf Phenolic Contents in Two Different Environments

**Figure 5:**
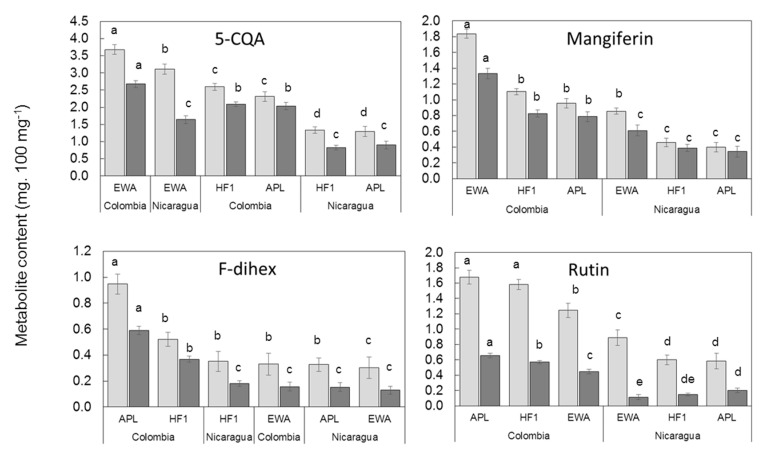
5-CQA, mangiferin, F-dihex and rutin averages of mature leaf content of trees grown in full sun (light gray) and under shade (dark gray) for Ethiopian wild accessions, American pure lines and F1 hybrid clones in Colombia and Nicaragua field trials. Different letters indicate significant differences at *p* < 0.05 (Newman–Keuls test) among means.

Next, we compared the leaf phenolic contents of the four compounds (5-CQA, mangiferin, F-dihex and rutin) that showed a significant correlation in the sun and in the shade, taking into account the averages calculated for each *C. arabica* genetic group, Ethiopian wild accessions, American pure lines and F1 hybrid clones, in Nicaragua and Colombia, in full sun and in the shade. As can be seen in Figure 5, whatever the compound and the location, the highest contents were observed in full sun. The difference between rutin contents in the sun and in the shade were particularly clear.

The concentration of 5-CQA in the leaves of the Ethiopian wild accessions was the highest under full sun regardless of the location of the trial, indicating that genotype dependence prevailed over location dependence. In the shade, concentrations of 5-CQA in the leaves of the Ethiopian wild accessions in Nicaragua were lower than those of all genetic groups in Colombia. Leaf contents in the shade were thus distributed according to the location and the relationship between genetic groups (Ethiopian wild accessions > F1 hybrid clones > American pure lines) was conserved.

The dependence on location of mangiferin and rutin contents in the leaves was clear, the samples from Colombia being separated from those from Nicaragua. The relationship between the concentration of mangiferin and the genetic group was conserved at the two locations regardless of the light condition (Ethiopian wild accessions > F1 hybrid clones > American pure lines) and the highest values were observed in Colombia. The same distribution was observed for rutin content, except for in the shade in Nicaragua, where the relationship between genetic groups was inversed, and the American pure lines had the highest rutin content (American pure lines > F1 hybrid clones > Ethiopian wild accessions).

Under each light condition, slight differences were observed between the concentration of F-dihex in all genetic groups in Nicaragua and the Ethiopian wild accession group in Colombia (Figure 5). It was thus impossible to identify a clear relationship between the F-dihex content and genetic group or location.

Interestingly, we observed that concentrations of 5-CQA, mangiferin and rutin in F1 hybrid clones grown in the sun were intermediate between those of their Ethiopian wild accession father and their American pure line mother, but closer to the latter. The same observation was made concerning concentrations of mangiferin and rutin in coffee trees grown in the shade.

## 3. Discussion

### 3.1. LC-MS^2^ Analysis Enabled Identification of Two Novel Mangiferin Derivatives in C. arabica

LC-DAD-MS^2^ was used for an exhaustive characterization of the alkaloids and phenolic compounds present in mature leaves of *C. arabica*. Two metabolites from the benzophenone group, an iriflophenone-C-glucoside and a xanthone derivative, mangiferin parahydroxybenzoate, were tentatively identified in coffee plants for the first time. Their presence in *C. arabica* leaves, along with confirmation of the presence of mangiferin previously described [31], points to the existence of a biosynthetic pathway for mangiferin via benzophenone synthase, as already reported in *Hypericum* and *Anemarrhena* [41,42] and reviewed in Joubert et al. [43].

The nature of the main accumulated flavanols was also clarified. They consist of catechin, epicatechin and a procyanidin. In contrast to the work of Ratanamarno and Surbkar [44], we detected no gallocatechin derivatives. Lastly, LC-MS^2^ analysis enabled annotation of some glycosylated derivatives of quercetin and kaempferol previously observed by Martins et al. [30]. These authors analyzed the phenolic content of the “Catuai Vermelho IAC 44” variety, an American pure line of *C. arabica*. Interestingly, the same 21 metabolites were identified in the mature leaves of the 34 genotypes analyzed in the present study, whether they were American pure lines, Ethiopian wild accessions or F1 hybrid clones, and irrespective of the light condition, elevation and latitude. However, some accumulated at concentrations that were too low to be quantified on HPLC-DAD chromatograms and only 16 could be used to compare the coffee leaf samples. In a quest for biochemical markers for use in cultivar development programs, it is preferable to target compounds that are relatively abundant and easy to quantify with simple instrumentation, ideally usable in field experiments.

### 3.2. Identification of Leaf Phenolic Contents as Biomarkers of Adaptation of C. arabica to Full-Sun or Shade Conditions

In agreement with the widely accredited role of phenolic compounds in the adaptive response of plants, particularly to abiotic environments [45,46,47], we were able to identify phenolics whose concentrations in *C. arabica* leaves changed with environmental conditions, especially with changes in light intensity, i.e., in our study, full sun vs. 50% shade.

#### 3.2.1. Preselection of Candidate Biomarkers of Adaptation of *C. arabica* to Full-Sun Conditions

Among the 16 phenolic compounds quantified by HPLC-DAD in the two field trails at two contrasting elevations in Mexico and in the study of a single American pure line (*C. arabica* cv. Marsellesa), we were able to identify compounds that could be used as markers of adaptation to full sun: 5-CQA, catechin, K-hex-dhex, F-dihex, rutin and mangiferin. These compounds were retained because their leaf contents were higher and/or displayed the highest significant difference between leaf contents in the sun and in the shade at at least one of the two elevations. Indeed, with the exception of K-hex-dhex, the compounds were present at medium to high concentrations in the leaves at least in full sunlight. K-hex-dhex was retained because of its highly significant differences in leaf contents in the sun and in the shade at both elevations. Additionally, the leaf contents of the six compounds were significantly correlated with those of the other compounds of the same chemical subgroup, thus reinforcing the results obtained, meaning they are good representatives of the response to light stress by these chemical subgroups.

#### 3.2.2. Selection of Biomarkers for Adaptation of *C. arabica* to Full Sunlight Using Genetic Diversity and Contrasting Environments

The six preselected candidate biomarkers for the adaptation of *C. arabica* to full sun were evaluated in seven other American pure lines, eight Ethiopian wild accessions and 19 of their F1 hybrids, in two contrasting geographical trials in Colombia and Nicaragua.

Combining the data on the concentration of each of the six preselected phenolics in the leaves of all 34 genotypes studied in the four field trials in Mexico, Colombia and Nicaragua, we finally retained four, 5-CQA, mangiferin, F-dihex and rutin, because the ratio of their concentration in the sun to that in the shade remained stable in the leaves of all the genetic material and in all four contrasting environments, as measured by *r*^2^ (0.74; 0.81; 0.75 and 0.63, respectively). All the slopes of the regression lines were below 1, indicating that, just like in cv. Marsellesa at two elevations in Mexico, the concentrations of these compounds in leaves of the 34 other genotypes were always higher in the sun than in the shade. The strong linear correlation between the concentrations of these four phenolics in the leaves suggests that *C. arabica* germplasm could be phenotyped in only one of the light conditions (full sun or 50% shade) but could still predict the degree of adaptation to full sunlight or shade, mimicking agroforestry systems. This also suggests that these four candidate biomarkers of adaptation could be used in very different environments and with contrasting genotypes like Ethiopian wild accessions and American pure lines.

#### 3.2.3. Influence of *C. arabica* Genotype on the Phenolic Leaf Contents and Selection of Biomarkers of Adaptation to Full Sunlight or Shade in Breeding Programs

By comparing the influence of the genotype on the four previously selected leaf phenolic contents at the two field trials in Colombia and Nicaragua, 5-CQA and mangiferin were shown to be the best candidate biomarkers of adaptation to full sunlight in *C. arabica* because both compounds globally reflected the genetic structure at the two locations and made it possible to differentiate Ethiopian wild accessions from American pure lines at the same location. Moreover, in the case of 5-CQA, the influence of genotype even prevailed over the influence of location, making it possible to differentiate Ethiopian wild accessions from American pure lines regardless of the location. All Ethiopian wild accessions consistently presented higher concentrations of 5-CQA in their leaves in the sun regardless of the location and of mangiferin at a same location compared to the American pure lines. This may be linked to the origin of the Ethiopian wild accessions, i.e., understory bushes in mesophilous forests [1] and that of American pure lines derived from the *C. arabica* “Yemen-Harare” group domesticated for cultivation in full sunlight [3,4,5,6,7,8,9].

The higher concentrations of both 5-CQA and mangiferin in leaves in full sun than in the shade observed in this study are in accordance with observations made in *C. arabica* cv. Catuai Vermelho IAC 44 in Brazil with two-fold higher accumulation of both compounds in leaves in full sun than in the shade [30]. Very little information is available on the likely role of 5-CQA in plant response to light stress, and mangiferin has been more widely studied in humans than in plants for its multipotent anti-inflammatory potential, anti-lipid peroxidation and antibacterial activities [48,49,50]. However, since this compound is considered to be a strongly antioxidant molecule, higher mangiferin contents in leaves exposed to full sunlight point to a likely role for mangiferin in protecting leaves from oxidative damage linked to more intense photosynthesis. A protective role of mangiferin against UV radiation in *Aphloia theiformis* has also been suggested [51]. A previous study on wild coffee species indicated higher accumulation of mangiferin in leaves of species originating from high altitudes where UV irradiance is higher [31]. It is interesting to note that, when we compared the average concentrations of 5-CQA and mangiferin in each genetic group (Ethiopian wild accessions, American pure lines and F1 hybrid clones), in the two trials, Colombia values were higher than Nicaragua values. This is consistent with the higher global horizontal irradiance observed during the trial in Colombia compared to the trial in Nicaragua (Table 3) and reinforces the capacity of these candidate biomarkers. However, the influence of other factors cannot be ruled out, e.g., that of UV-B irradiance, which increases as one approaches the Equator, or of temperature which, in our case, was higher in Colombia.

An interesting result concerning 5-CQA was that, in the two field trials in Mexico, we found a significant correlation between the concentration of 5-CQA in leaves and the concentrations of all the other phenolic compounds analyzed, except quercetin derivatives. This suggests that 5-CQA plays a key role in the response of phenolic metabolism to light stress, as suggested by Grace and Logan [17]. Phenylpropanoid biosynthesis may offer an alternative pathway for photochemical energy dissipation. In this energy overflow mechanism, which transforms photosynthesis products into stable compounds, 5-CQA may be the main sink.

Another important observation is that in both the Nicaragua and Colombia trials, the F1 hybrid clones had very similar concentrations of 5-CQA and mangiferin in the leaves of shaded and unshaded plants to those measured in their mother American pure lines, suggesting a significant maternal effect in adaptation to light growing conditions. This should be taken into account in future breeding programs.

The concentrations of 5-CQA and mangiferin in leaves in full sun and in the shade are among the highest observed for the phenolic compounds, irrespective of the light conditions and the field trial, particularly the concentration of 5-CQA. Thus, their detection and quantification are quite easy, accurate and reproducible.

5-CQA is the most common chlorogenic acid in plants. As an esterified form of caffeic acid with quinic acid, this compound possesses a phenol ring produced by hydroxycinnamic acid and a specific absorption spectrum. Near-infrared (NIR) spectroscopy has been successfully used to characterize coffee beans and to study environmental effects on coffee bean quality [52,53]. An NIR calibration was obtained, based on partial least squares regression with quantitative values of different concentrations of CQA [54]. The NIR technique thus enables rapid evaluation of CQA content in coffee beans. More recently, a study was conducted on coffee leaves using Fourier transform near infrared spectroscopy (FT-NIR) combined with a statistical method for classification (SIMCA) [55]. Mangiferin also possesses a phenol ring and a specific absorption spectrum. Thus, CQA and mangiferin evaluation by NIR could be considered for high-throughput phenotyping of *C. arabica* germplasm. This approach would help identify candidate progenitors with good adaptation to full-sun conditions, i.e., likely those with low leaf 5-CQA and mangiferin contents, or to shaded agroforestry conditions, i.e., those with high leaf 5-CQA and mangiferin contents. It would facilitate high-throughput phenotyping of large F1 offspring and the creation of new F1 hybrids.

Like 5-CQA and mangiferin, the concentration of F-dihex in full sun was higher than in the shade in all the genotypes studied. However, although the concentration of F-dihex in leaves in full sun was well correlated with the concentration in the shaded leaves across the different genotypes and the four field trials, this compound did not reflect genetic structure (Ethiopian wild accessions vs. American pure lines) when its leaf contents were compared in different genetic groups, meaning F-dihex cannot be used as a stable biomarker of the adaptation of *C. arabica* to full sunlight.

Rutin leaf contents were considerably higher in plants grown in full sun than in plants grown under shade in all the genotypes studied and at all four study locations. This is in agreement with the results of previous studies showing that quercetin derivatives [56] and, more globally, flavonols [57] are involved in plant response to light, particularly to UV-B radiation. This could explain why concentrations of rutin in the leaves of *C. arabica* cv. Marsellesa in Mexico in both the sun and shade were much higher at the high elevation (1250 m asl) than at the low elevation (650 m asl), as global horizontal irradiance was very similar at the two trial sites but UV-B radiation increases with elevation. The marked variations in rutin contents in the same variety grown at two contrasting elevations points to high dependence of the compound biosynthesis on environmental conditions. Moreover, when comparing rutin content between genetic groups, the relationship between concentrations in leaves grown in the sun and in the shade did not reflect the genetic structure of the genotypes studied, i.e., Ethiopian wild accessions vs. American pure lines. Thus, rutin appears to be of no interest as a selection biomarker for adaptation to growth in full sun, but could be useful to assess light stress, particularly UV stress, in a given coffee genotype at a given time and in different environmental conditions. This is in accordance with the stimulation of quercetin derivative biosynthesis by light intensity observed in coffee leaves under natural [30] or controlled conditions [58] and in line with the reaction of numerous plants to UV-B irradiance [56]. Recent studies indicated that quercetin derivatives, and more specifically ortho-dihydroxylated B-ring flavonoids, play an important role in photoprotection as ROS-detoxifying agents and, to a lesser extent, as UV screens [59,60,61].

## 4. Materials and Methods

### 4.1. Locations and Plant Material

**Figure 6:**
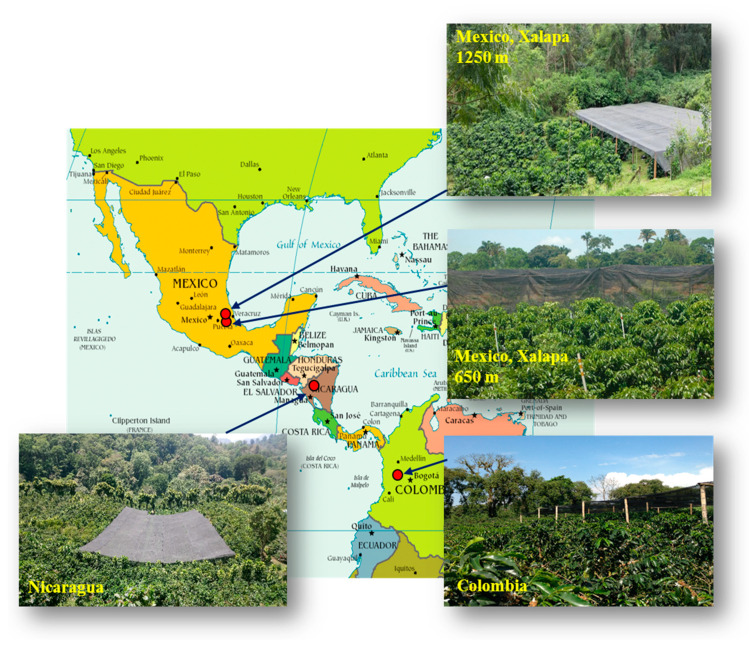
Geographical distribution of the four experimental sites in America: Mexico (two sites at different elevations), Nicaragua and Colombia. In each site, half of the *C. arabica* trees are under shade.

This study was conducted at four locations in three countries, two in Mexico, one in Nicaragua and one in Colombia, from latitude 4° N to 19° N, which covers most of the Latin American *C. arabica* growing area in the Northern Hemisphere (Figure 6). Table 3 lists the location and main climatic and agronomic factors at each location. Each field trial was made up of two adjacent twin plots, each including all the genotypes studied, one in full sunshine and the other one under a black polyethylene shading net that excluded 50% of the light. When the leaf samples were collected, the coffee trees were three years old in Mexico and Colombia. In Nicaragua, they were also 3 years old after the coppicing of the original 24-year-old trees. The coffee trees were considered to be adults with full fruit production at all the elevations of the four experimental field trails. In Mexico, the study was carried out in two similar field trials located at two contrasting elevations (650 m vs. 1250 m asl) to generate a proxy of temperatures in order to mimic global warming associated with climate change.

In the two experimental plots in Mexico, a single American pure line of *C. arabica* was studied: cv. Marsellesa, belonging to the Sarchimor group (a cross between the Timor hybrid CIFC 832/2, a natural interspecific hybrid of *C. arabica* and *C. canephora*) and the Costa Rican compact line Villa Sarchi). The 22 genotypes studied in Nicaragua and the 11 studied in Colombia were *C. arabica* American pure lines, Ethiopian wild accessions of *C. arabica* and cloned F1 hybrids propagated by somatic embryogenesis, and from crosses between American pure lines as mothers and Ethiopian wild accessions as fathers, except for two F1 hybrid clones in the Nicaraguan trial that were crosses between two American pure lines. Table 4 lists the details of all the plant material studied in the three countries.

### 4.2. Leaf Sampling for Secondary Metabolite Analysis

For each growth condition (full sun or shade), at between 5 and 6 h after sunrise, ten mature leaves were collected from the same tree at the third node (from the branch apex) of the 6th to 8th pairs of plagiotropic branches (counting from the orthotropic apex). For samples of plants growing in full sun, care was taken to ensure that the leaves collected were fully exposed to the sun (by avoiding shaded leaves) and faced eastward. Leaves from the same tree grown in the same conditions were packed together and immediately plunged into liquid nitrogen and conserved at −80 °C until freeze dried. For each growth condition, 9 to 10 trees were sampled in June 2015 in Mexico, three to five trees in April 2019 in Nicaragua, and two to six trees (two American pure lines, three Ethiopian wild accessions and six F1 hybrid clones) in June 2014 in Colombia.

### 4.3. Secondary Metabolite Analysis

The freeze-dried leaf samples were vacuum packed and sent to the IRD laboratory, Montpellier, France, where they were ground into a fine powder in an A10 IKA model electric blender (IKA^®^-Werke GmbH & Co. KG, Staufen, Germany) and stored until extraction. Each sample was extracted by stirring 25 mg of plant material in 6 mL of MeOH/H_2_O (80:20, *v*/*v*) in a Rotamax 120 (Heidolph Instruments GmbH & CO. KG, Schwabach, Germany) at 250 rpm and at 4 °C for 3 h, supplemented with 10 µL of 5-methoxyflavone (4 mM) as an internal standard. After centrifugation at 3500 rpm at 8 °C for 10 min, the organic extract was collected and filtered (Millipore, 0.25 µm porosity) before analysis. Each extraction was carried out in triplicate.

Quantitative analyses were carried out on a Shimadzu LC 20 HPLC-DAD system (Shimadzu Corporation, Kyoto, Japan) as described by Campa et al. [58]. Parallel analyses were performed in triplicate on pure standard solutions of trigonelline, theobromine, caffeine, mangiferin, 5-CQA and caffeic acid purchased from Sigma-Aldrich Chimie (St Quentin Fallavier, France), while glycosylated kaempferols (kaempferol and kaempferol-3-*O*-glucoside), quercetin, rutin, (+)-catechin, (-)-epicatechin and epigallocatechin came from Extrasynthese (Lyon, France) and 3,4-, 3,5- and 4,5-*O*-dicaffeoylquinic acid (diCQA) came from Biopurify Phytochemicals (Chengdu, China) at 25, 50, 75 and 100 µg.mL^−1^. Quantification of 3-, 4- and 5-CQA, FQAs and 3,4-, 3,5- and 4,5-diCQA was undertaken at 320 nm, caffeine and catechin derivatives at 280 nm and mangiferin, kaempferol and quercetin derivatives at 360 nm. Concentrations are expressed in mg.100 mg^−1^ dry weight (DW) by comparison with standard curves. 3-CQA, 4-CQA and FQA contents were calculated relative to the 5-CQA standard curve. Concentrations of flavonol derivatives except rutin were calculated based on the standard quercetin curve, and are expressed as quercetin equivalent. Each sample of coffee leaf powder was extracted and analyzed three times. The mean concentrations of phenolic compounds are expressed in mg.100 mg^−1^ DW.

To confirm the standard-based identifications and to provide some basic structural information on the other substances, LC-DAD-MS^2^ analyses were performed on an Agilent Infinity^®^ 1290 system (Agilent Technologies, Santa Clara, USA) coupled to a UV/vis DAD detector and equipped with a QTOF 6530 detector (Agilent) controlled by MassHunter^®^ software (Agilent). Analytical separation was carried out on a Poroshell^®^ 120 EC-C18 column (100 mm × 3.0 mm, 2.7 μm) equipped with a pre-column (Poroshell^®^ 20 EC-C18, 5 mm × 3.0 mm, 2.7 µm). A gradient of 0.4% formic acid in water (A) and acetonitrile (B) was used as follows: 0 min, 1% B; 1.5 min, 1% B; 6 min, 10% B; 12 min, 35% B; 14 min, 100% B; 16 min, 100% B. The flow rate and column temperature were 1.0 Ml.min^−1^ and 60 °C, respectively. A 2.0 µL aliquot of sample extract was injected into the column. The ESI source was optimized for positive and negative ionization modes (in “Auto MSMS” acquisition mode) as follows: scan spectra from *m*/*z* 50 to 2000, capillary voltage 3.5 kV, nozzle voltage 2000 V, fragmentor 110 V, fixed collision-induced dissociation (CID) energy at 20 eV. Nitrogen was used as the nebulizing gas at a flow rate of 12 L.min^−1^ and a temperature of 310 °C at 40 psi.

### 4.4. Statistical Analysis

All statistical analyses were performed in Statistica^®^ 7.1. Biochemical data were checked for homogeneity of variance using Levene’s test. A factorial analysis of variance (ANOVA) with a Newman–Keuls test (*p* < 0.05) for comparing averages was performed on the 16 quantified phenolic contents for samples from Mexico (elevation × growing light condition). A Pearson’s correlation matrix was also performed for leaf contents of these same 16 phenolic compounds by pooling sun and shade data. A heatmap was constructed with R software using the gplots package with default parameters for symmetrical dendrogram computation (linkage clustering using the Euclidian distance measures) and reordering using row (equal to column) means and a linear set of 50 shades of color (no color break) as correlation coefficient bins [62]. A linear regression analysis was performed on six phenolic leaf contents (5-CQA, catechin, mangiferin, K-hex-dhex, F-dihex and rutin), pooling all studied genotype samples from Colombia and Nicaragua. A Newman–Keuls test (*p* < 0.05) was performed for 5-CQA, mangiferin, F-dihex and rutin leaf contents on averages of combinations of genetic groups (Ethiopian wild accessions vs. American pure lines vs. F1 hybrid clones) × country (Colombia vs. Nicaragua).

## 5. Conclusions

This study allowed us to identify two phenolic compounds, chlorogenic acid 5-CQA and xanthone mangiferin, as good candidate biomarkers of *C. arabica* adaptation to variations in light intensity. Analysis of concentrations of the two compounds in the leaves of 34 *C. arabica* genotypes belonging to three different genetic groups grown in full sun or under 50% shade showed that the best adapted genotypes to high light intensity had the lowest 5-CQA and mangiferin contents, a trait that was preserved when grown in the shade. The predictive accuracy of these two biomarkers should now be confirmed on a wider range of *C. arabica* genotypes among the recently identified genetic groups of wild *C. arabica* germplasm. The aim would be to confirm our hypothesis that high 5-CQA and/or mangiferin leaf contents are a signature of greater adaptation to shade, i.e., to agroforestry systems, and conversely, that low concentrations of the compounds in leaves indicate good adaptation to full-sun cropping systems. Although the two compounds are good biomarkers for the degree of *C. arabica* adaptation to light, under no circumstances do they explain the mechanisms of *C. arabica* genotype adaptation to full sunlight. Questions concerning the molecular basis of the adaptation of arabica trees to full-sunlight conditions remain unanswered. In addition, proven analytical techniques that allow fast, accurate and reliable dosages of these two compounds, such as NIR spectroscopy techniques, make it possible to envisage opportunities for high-throughput phenotyping of *C. arabica* genotypes.

## Figures and Tables

**Table 1:** Result of the LC-DAD-MS^2^ analysis performed with a leaf methanolic extract of *C. arabica* cv. Marsellesa. RT, retention time in the analytic system in minutes; Mass, theoretical mass expressed in Da; Wavelength, maximum absorbance in the UV range; sh, shoulder. Experimental mass accuracy of less than 2 ppm. ND: not detected; *: metabolites identified by comparison with a pure standard compound.

Peak N°	RT (min)	Mass (Da)	Molecular Formula	Wavelength (nm)	Compound Name	[M − H]^−^	MS^2^	[M+H]^+^	MS2
1	2.61	180.1640	C_7_H_8_N_4_O_2_	272	Theobromine *	-		181.0718	67 (100) 85 (78) 181 (74) 138 (65) 69 (61) 108 (61) 56 (43) 163 (43) 122 (28) 117 (13) 156 (9)
2	3.00	354.3087	C_16_H_18_O_10_	300sh-325	3-*O*-Caffeoylquinic acid	353.0901	191 (100) 179 (61) 135 (47) 173 (6)	355.1002	163 (100) 145 (24) 89 (9)
3	4.02	408.3561	C_19_H_20_O_10_	286	Iriflophenone-*C*-hexose	407.1012	287 (100) 317 (23) 245 (61) 193 (47) 125 (8)	409.1107	195 (100) 231 (79) 13 (61) 325 (46) 177 (43) 121 (39) 271 (39) 355 (14) 85 (7)
4	4.20	290.2700	C_15_H_14_O_6_	278	(+)-Catechin *	289.0769	109 (100) 123 (69) 125 (64) 203 (56) 151 (44) 245 (40) 137 (36) 205 (26) 97 (25) 121 (23) 289 (21) 187 (21) 188 (20) 149 (20) 221 (20) 161 (20)	291.0858	139 (100) 123 (59) 147 (23) 161 (12) 207 (6)
5	4.42	354.3087	C_16_H_18_O_10_	300sh-325	5-*O*-Caffeoylquinic acid *	353.0901	191 (100) 161 (2) 179 (2) 173 (1)	355.1002	163 (100) 145 (11) 164 (11) 135 (4) 117 (3) 89 (2)
6	4.74	354.3087	C_16_H_18_O_10_	300sh-325	4-*O*-Caffeoylquinic acid	353.0901	173 (100) 191 (85) 179 (73) 135 (51) 93 (20)	355.1002	163 (100) 145 (14) 164 (9) 117 (6) 135 (5) 89 (4)
7	4.90	194.1900	C_8_H_10_N_4_O_2_	272	Caffeine *	-		195.089	138 (100) 110 (36) 195 (29) 69 (21)
8	5.42	290.2700	C_15_H_14_O_6_	278	(-)-Epicatechin *	289.0709	109 (100) 125 (74) 203 (69) 123 (60) 151 (43) 245 (40) 137 (37) 205 (34) 161 (26) 97 (23) 188 (23) 221 (22) 187 (20) 121 (20) 179 (20) 289 (19)	191.0844	139 (100) 123 (63) 147 (24) 161 (12) 207 (6)
9	5.62	338.3093	C_16_H_18_O_8_	310	5-*O*-Coumaroylquinic acid	337.0953	191 (100) 133 (17) 53 (12) 93 (12) 253 (12) 145 (10) 75 (2) 302 (2)	339.1085	147 (100) 75 (23) 97 (23) 236 (23) 127 (19) 258 (16) 341 (16) 65 (10) 189 (10) 217 (10) 306 (10)
10	6.08	422.3400	C_19_H_18_O_11_	257-317-365	Mangiferin *	421.0799	301 (100) 331 (85) 259 (12) 421 (15) 273 (10)	423.0906	273 (100) 303 (74) 327 (51) 369 (22) 351 (18) 299 (15) 357 (9)
11	6.79	368.3353	C_17_H_20_O_9_	258-267sh-300sh-327	5-*O*-Feruloylquinic acid	367.1058	191 (100) 93 (21) 173 (11) 111 (7) 134 (4)	369.1157	177 (100) 145 (42) 117 (5)
12	6.81	594.5181	C_27_H_30_O_15_	256(sh)-265-330	Flavone di-C-hexose	593.1555	593 (100) 473 (11) 353 (10) 406 (10) 503 (8)	595.1643	457 (100) 427 (67) 379 (35) 325 (29) 295 (12)
13	6.86	772.6581	C_33_H_40_O_21_	257-300sh-353	Quercetin-3-*O*-dihexose-deoxyhexose	771.2036	771 (100) 300 (53) 179 (5) 273 (5) 271 (5)	773.2129	303 (100) 125 (53) 84 (26) 194 (13)
14	6.97	626.5169	C_27_H_30_O_17_	257-300sh-353	Quercetin-3,4-di-*O*-glucoside *	625.1449	625 (100) 300 (72) 271 (10) 179 (3) 445 (3) 463 (3) 505 (3)	627.1514	303 (100)
15	7.34	756.659	C_33_H_40_O_20_	265-346	Kaempferol-3-*O*-dihexose-deoxyhexose	755.2128	ND	757.2131	287 (100) 449 (6) 129 (6) 757 (6) 612 (1)
16	7.74	610.5175	C_27_H_30_O_16_	257-353	Quercetin-3-*O*-rutinoside *	609.1539	609 (100) 300 (20)	611.1595	303 (100)
17	7.90	578.5202	C_30_H_26_O_12_	276	Procyanidin dimer	577.1396	289 (100) 577 (83) 407 (72) 125 (61) 161 (17) 451 (11)	579.1472	287 (100) 409 (94) 127 (84) 580 (64) 163 (31) 247 (19) 579 (13)
18	7.96	464.3800	C_21_H_20_O_12_	257-353	Quercetin-3-*O*-glucoside *	463.0908	300 (100) 463 (48) 191 (19) 271 (10)	465.1018	303 (100)
19	8.20	594.5181	C_27_H_30_O_15_	265-346	Kaempferol-3-*O*-hexose-deoxyhexose	593.1540	ND	595.1629	287 (100) 596 (1)
20	8.39	516.451	C_25_H_24_O_12_	246-300sh-324	3,4-*O*-Dicaffeoylquinic acid *	515.1221	173 (100) 353 (95) 179 (70) 191 (34) 354 (21) 515 (21) 335 (14)	517.1323	163 (100) 63 (27) 140 (18) 191 (18) 370 (11)
21	8.48	516.451	C_25_H_24_O_12_	243-297sh-327	3,5-*O*-Dicaffeoylquinic acid *	515.1229	191 (100) 353 (91) 179 (63) 135 (13) 173 (6) 515 (3)	517.1323	140 (100) 163 (77) 519 (64) 213 (59) 117 (41) 303 (41) 189 (27) 238 (27) 367 (27) 442 (27) 492 (23)
22	8.93	516.4510	C_25_H_24_O_12_	243-297sh-327	4,5-*O*-Dicaffeoylquinic acid *	515.1229	353 (100) 173 (92) 179 (53) 354 26) 191 (16) 135 (11) 137 (7) 203 (6)	517.1323	163 (100) 111 (18) 383 (14) 435 (14) 224 (5)
23	9.49	542.4451	C_26_H_22_O_13_	257-317-365	Mangiferin parahydroxybenzoate	541.1017	301 (100) 331 (79) 541 (53) 259 (6) 385 (6)	543.1118	303 (100) 121 (96) 405 (24) 327 (23) 543 (21)

**Table 2:** Factorial ANOVA of the concentrations of phenolic compounds in the leaves of *C. arabica* cv. Marsellesa according to elevation (650 m asl vs. 1250 m asl) and light conditions (full sun vs. 50% shade). For each chemical family, the compound(s) with the most significant difference in leaf contents for one or both environmental factors and the corresponding *F* score are underlined.

Phenolic Compounds	Light		Elevation		Elevation x Light	
	*F*	*p*	*F*	*p*	*F*	*p*
**Chlorogenic Acids**						
3-CQA	1.72	0.198936	7.72	0.008836	0.00	0.954559
4-CQA	0.05	0.823017	29.29	0.000005	5.81	0.021526
5-CQA	18.69	0.000127	56.51	0.000000	28.72	0.000006
3,4-diCQA	1.97	0.169890	35.48	0.000001	11.11	0.002085
3,5-diCQA	6.88	0.012958	4.77	0.035947	11.71	0.001633
4,5-diCQA	8.20	0.007112	49.98	0.000000	35.19	0.000001
FQA	0.03	0.865203	0.23	0.633213	4.21	0.048007
**Flavonoids**						
Flavanols						
Epicatechin	6.00	0.019604	63.86	0.000000	10.29	0.002912
Catechin	0.13	0.717829	100.22	0.000000	23.80	0.000025
Glycosylated flavonoids						
K-dihex-dhex	26.33	0.000012	29.04	0.000005	15.76	0.000353
K-hex-dhex	117.43	0.000000	23.92	0.000024	29.81	0.000004
F-dihex	16.15	0.000307	13.02	0.000980	8.79	0.005506
Q-dihex-dhex	54.52	0.000000	11.64	0.001685	0.30	0.589041
Q-diGlu	100.65	0.000000	23.18	0.000030	0.01	0.903435
Rutin	142.72	0.000000	31.26	0.000003	0.02	0.881375
**Xanthones**						
Mangiferin	31.19	0.000003	12.69	0.001112	2.39	0.131611

**Table 3:** Location, main climate data and cropping conditions in the four experimental field trials in Mexico, Nicaragua and Colombia.

Country	Geographic Coordinates	Elevation (m asl)	Plant Density (m)	Plants for Each Half Plot: Full Sun or under Shade (N)	Annual Average Temperature Min/Max (°C)	Average Surface Temperature (°C)	Rain (mm by Year)	Global Horizontal Irradiation (kWh.m^−2^)
Mexico	18°51′46″ N 96°51′35″ W	650	2 × 1.5	192	18.6/31.4	19.1	2650	1890
Mexico	19°23′43″ N 96°59′60″ W	1250	2 × 1.5	192	14.2/28.2	17.0	2100	1848
Nicaragua	13°2′45″ N 85°53′30″ W	1200	2 × 1	950	14.1/24.0	18.7	1760	1706
Colombia	4°58′17″ N 75°39′09″ W	1380	1.3 × 1.5 for APL 1.5 × 1.5 for WEA	490	17.4/26.8	22.1	3570	1851

APL: American pure lines; EWA: Ethiopian wild accession.

**Table 4:** Plant material used in the experimental plots in Mexico, Nicaragua and Colombia. APL, American pure lines; EWA, Ethiopian wild accessions.

Genotype	Genealogy	Origin	Genetic Group
**Mexican experimental plot**			
Marsellesa^®^	Timor hybrid CIFC 832/2 x cv. Villa Sarchi (Costa Rica)	CIRAD Nicaragua	APL of Sarchimor group
**Nicaraguan experimental plot**			
ET08 A8	EWA	Kaffa province, Ethiopia (ORSTOM prospecting, 1966)	“Jimma Bonga” (G1A) *
ET47 A4		Kaffa province, Ethiopia (FAO prospecting, 1964–1965)	
ET26 A1			“Sheka” (G1B) *
ET25 A4			G1G2 *
ET06			Not yet characterized
T5175	Timor hybrid CIFC 832/1 x cv. Caturra	*Instituto del Café* of Costa Rica (ICAFE)	APL of Catimor group
T8667	Timor hybrid CIFC 832/1 x cv. Caturra	CATIE, Turrialba, Costa Rica	
T5296	Timor hybrid CIFC 832/2 x cv. Villa Sarchi		APL of Sarchimor group
T17931	Timor hybrid CIFC1343 x cv. Caturra		APL Catimor line of the multiline var. Colombia
Catuaí	cv. Mundo Novo x cv. Caturra	*Instituto Agronômico de Campinas* (IAC), Brazil	APL non-introgressed dwarf cultivar
T5175 x ET08 A8	APL mother x EWA father F1 hybrid	CIRAD, Nicaragua	F1 hybrid clone
T5175 x ET26 A1			
T5175 x ET25 A4			
T5175 x T17931	APL mother x APL father F1 hybrid		
T8667 x ET47 A4	APL mother x EWA father F1 hybrid		
T8667 x ET26 A1			
T8667 x T5296	APL mother x APL father F1 hybrid		
T5296 x T17931			
T17931 x ET47 A4	APL mother x EWA father F1 hybrid		
T17931 x ET26 A1			
T17931 x ET25 A4			
Catuaí x ET47 A4			
Catuaí x ET26 A1			
**Colombian experimental plot**			
E554	EWA	Kaffa province, Ethiopia (FAO 1964-1965)	“Jimma Bonga” (G1A) *
E286		Kaffa province, Ethiopia (FAO 1964-1965)	“Jimma Bonga” (G1A) *
E057		Kaffa province, Ethiopia (FAO 1964-1965)	“Jimma Bonga” (G1A) *
CX2385	Timor hybrid CIFC1343 x cv. Caturra	CENICAFE, Colombia	APL of Catimor group
CU1842			
CX2385 x E554	APL mother x EWA father F1 hybrid		F1 hybrid clone
CX2385 x E286			
CX2385 x E057			
CU 1842 x E554			
CU 1842 x E286			
CU 1842 x E057			

* Genetic groups of Ethiopian wild accessions (EWA) according to Scalabrin et al. [3].

## Supplementary Materials

The following are available online at https://www.mdpi.com/2218-1989/10/10/383/s1, Table S1: Concentrations of phenolic compounds in mature leaves of *C. arabica* cv. Marsellesa grown at two different elevations (650 and 1250 m asl) in full sun vs. under 50% shade, Table S2: Concentrations of six major phenolic compounds in mature leaves of *C. arabica* grown in Nicaragua and Colombia in full sun or under 50% shade.

## Abbreviations

Amu	atomic mass unit
APL	American pure lines
CGAs	chlorogenic acid group (CQA, diCQA, Coum-QA and FQA)
Coum-QA	coumaroyl quinic acid
3-CQA	3-*O*-caffeoylquinic acid
4-CQA	4-*O*-caffeoylquinic acid
5-CQA	5-*O*-caffeoylquinic acid
3,4-diCQA	3,4-*O*-dicaffeoylquinic acid
3,5-diCQA	3,5-*O*-dicaffeoylquinic acid
4,5-diCQA	4,5-*O*-dicaffeoylquinic acid
EWA	Ethiopian wild accessions
F-dihex	flavone-di-C-hexose
FQA	feruloylquinic acid
HF1	hybrid F1 clone
HPLC-DAD	High performance liquid chromatograph/ photodiode array detection
K-dihex-dhex	kaempferol-3-*O*-di-hexose-deoxyhexose
K-hex-dhex	kaempferol-3-*O*-hexose-deoxyhexose
LC-DAD-MS^2^	High performance liquid chromatograph/ photodiode array detection /mass spectrometry^2^
*p*OH Mang	mangiferin parahydroxybenzoate
Q-dihex-dhex	quercetin-3-*O*-dihexose-deoxyhexose
Q-diGlu	quercetin-3-*O*-diglucoside
SNP	single-nucleotide polymorphism
