# Supplementary material for: 5-CQA and Mangiferin, Two Leaf Biomarkers of Adaptation to Full Sun or Shade Conditions in Coffea arabica L
Source: Metabolites. 2020 Sep 26;10(10):383. doi: 10.3390/metabo10100383 (PMC7599603; doi:10.3390/metabo10100383)
Supplement: Supplementary file 1 [file metabolites-10-00383-s001.pdf]

Table S1. Content in phenolic compounds of mature leaves of *C. arabica* cv. Marsellesa grown at two different elevations (650 and 1250 m asl) and in full-sun *vs* under shade (means and standard deviation of ten and nine plants, respectively). Different letters indicate significant differences at $p < 0.05$ (Newman–Keuls test) among means.

Compounds		Content (mg.100^{-1} mg DW leaves)			
		Low elevation		High elevation	
		Full sun	Shade	Full sun	Shade
**Chlorogenic acids**	3-CQA	0.06 ± 0.01 *a*	0.05 ± 0.02 *ab*	0.04 ± 0.01 *ab*	0.04 ± 0.01 *b*
	5-CQA	4.83 ± 0.57 *a*	3.45 ± 0.36 *b*	3.00 ± 0.43 *b*	3.15 ± 0.32 *b*
	4-CQA	0.19 ± 0.03 *a*	0.17 ± 0.03 *a*	0.12 ± 0.03 *b*	0.15 ± 0.01 *b*
	3,4-diCQA	0.04 ± 0.01 *a*	0.03 ± 0.01 *b*	0.01 ± 0.01 *c*	0.03 ± 0.01 *bc*
	3,5-diCQA	0.12 ± 0.04 *a*	0.11 ± 0.01 *a*	0.07 ± 0.03 *b*	0.12 ± 0.01 *a*
	4,5-diCQA	0.04 ± 0.01 *a*	0.02 ± 0.01 *b*	0.01 ± 0.01 *c*	0.02 ± 0.01 *b*
	FQA	0.07 ± 0.02 *a*	0.05 ± 0.03 *a*	0.05 ± 0.03 *a*	0.06 ± 0.01 *a*
**Flavonoids**	F-dihex	0.60 ± 0.18 *a*	0.33 ± 0.10 *b*	0.34 ± 0.11 *b*	0.30 ± 0.01 *b*
	Q-dihex-dhex	0.04 ± 0.01 *b*	0.02 ± 0.01 *c*	0.05 ± 0.01 *a*	0.03 ± 0.01 *c*
Glycosylated flavonoids	Q-diGlu	0.18 ± 0.04 *b*	0.07 ± 0.01 *d*	0.23 ± 0.03 *a*	0.12 ± 0.03 *c*
	K-dihex-dhex	0.03 ± 0.01 *a*	0.01 ± 0.00 *b*	0.01 ± 0.00 *b*	0.00 ± 0.00 *b*
	K-hex-dhex	0.04 ± 0.01 *a*	0.01 ± 0.00 *c*	0.02 ± 0.00 *b*	0.01 ± 0.00 *c*
	Rutin	0.70 ± 0.19 *b*	0.16 ± 0.09 *d*	0.95 ± 0.12 *a*	0.42 ± 0.12 *c*
Flavanols	Catechin	2.27 ± 0.23 *a*	1.87 ± 0.22 *b*	1.48 ± 0.21 *d*	1.16 ± 0.20 *c*
	Epicatechin	1.52 ± 0.09 *a*	1.47 ± 0.23 *a*	0.79 ± 0.25 *c*	1.16 ± 0.20 *b*
**Xanthones**	Mangiferin	1.69 ± 0.34 *a*	1.16 ± 0.11 *bc*	1.31 ± 0.23 *b*	1.01 ± 0.12 *c*

Table S2. Content in six major phenolic compounds of mature leaves of *C. arabica* grown in Nicaragua and Colombia under full sun or shade condition.

**Country**	**Genetic group**	**Genotype**	**5-CQA**		**Catechin**		**K-hex-dhex**		**F-dihex**		**Rutin**		**Mangiferin**	
			**Sun**	**Shade**	**Sun**	**Shade**	**Sun**	**Shade**	**Sun**	**Shade**	**Sun**	**Shade**	**Sun**	**Shade**
Nicaragua	APL (mothers)	T5175	2.09	1.18	1.68	1.35	0.05	0.01	0.41	0.17	0.75	0.26	0.50	0.35
		T8667	1.53	0.99	1.09	1.11	0.05	0.01	0.41	0.14	0.83	0.29	0.38	0.30
		T5296	1.45	1.21	1.17	1.60	0.02	0.01	0.29	0.17	0.32	0.14	0.54	0.63
		Catuai	0.97	0.41	1.31	0.79	0.06	0.00	0.26	0.07	0.72	0.13	0.42	0.20
		T17931	0.46	0.71	0.47	1.45	0.03	0.02	0.27	0.22	0.31	0.19	0.17	0.23
	EWA (fathers)	ET25	2.97	1.87	1.54	1.64	0.11	0.06	0.06	0.03	0.49	0.15	1.21	1.13
		ET06	2.93	1.79	1.95	1.70	0.01	0.01	0.40	0.19	0.90	0.11	0.68	0.61
		ET26	3.95	1.58	1.91	1.62	0.05	0.02	0.37	0.19	1.37	0.11	0.82	0.64
		ET08	2.83	1.62	1.36	0.86	0.09	0.08	0.23	0.03	0.76	0.05	0.70	0.17
		ET47	2.90	1.35	1.65	1.25	0.04	0.02	0.46	0.20	0.92	0.15	0.86	0.51
	HF1	T5175 * ET25	1.64	0.84	1.84	2.05	0.06	0.02	0.28	0.14	0.51	0.13	0.66	0.61
		T8667 * ET26	1.93	1.06	1.36	1.18	0.09	0.04	0.38	0.26	0.60	0.17	0.53	0.52
		T8667 * ET47	1.34	0.67	1.62	1.02	0.06	0.01	0.41	0.16	0.59	0.07	0.51	0.27
		Catuai * ET26	1.13	0.54	1.27	0.90	0.04	0.02	0.25	0.13	0.48	0.07	0.32	0.26
		Catuai * ET47	2.45	1.37	2.04	1.89	0.05	0.01	0.37	0.19	0.56	0.09	0.83	0.58
		T5175 * ET26	1.55	0.87	1.64	1.69	0.05	0.01	0.41	0.22	0.73	0.12	0.46	0.41
		T17931 * ET26	0.78	0.79	0.96	0.87	0.05	0.03	0.36	0.24	0.60	0.23	0.31	0.41
		T5175 * ET08	2.37	0.97	1.72	1.24	0.04	0.01	0.38	0.15	1.01	0.18	0.74	0.38
		T17931 * ET47	0.65	0.59	0.87	0.83	0.06	0.03	0.34	0.25	0.47	0.28	0.25	0.17
		T17931 * ET25	0.68	0.76	1.03	1.49	0.04	0.03	0.19	0.09	0.31	0.11	0.35	0.44
		T5175 * 17931	0.93	0.91	1.20	1.48	0.04	0.01	0.42	0.16	0.60	0.15	0.28	0.36
		T8667 * T5296	0.60	0.55	0.73	0.83	0.04	0.01	0.34	0.19	0.60	0.12	0.25	0.23
		T5296 * 17931	1.35	0.83	1.19	1.56	0.05	0.01	0.45	0.16	0.73	0.19	0.47	0.42
Colombia	APL (mothers)	CX2385	1.99	1.85	1.66	1.54	0.08	0.02	0.95	0.53	1.40	0.59	0.75	0.65
		CU1842	2.64	2.22	1.98	2.08	0.07	0.02	0.95	0.65	1.96	0.73	1.16	0.92
	EWA (fathers)	E057	3.82	2.54	1.33	1.31	0.08	0.06	0.42	0.25	1.31	0.58	1.34	1.05
		E286	3.63	2.86	0.97	1.30	0.25	0.17	0.26	0.12	1.10	0.44	1.40	1.33
		E554	3.60	2.64	0.91	0.85	0.30	0.04	0.31	0.10	1.32	0.32	2.77	1.62
	HF1	CX2385 x E286	2.51	2.37	1.16	2.06	0.12	0.06	0.34	0.27	1.35	0.67	0.96	0.93
		CX2385 x E057	2.49	2.08	1.53	1.01	0.13	0.03	0.39	0.36	1.75	0.49	1.04	0.75
		CX2385 x E554	2.33	1.79	2.01	1.82	0.14	0.02	0.95	0.57	1.81	0.46	1.04	0.62
		CU1842 x E286	2.80	2.21	1.47	2.09	0.11	0.05	0.45	0.31	1.45	0.70	1.01	0.92
		CU1842 x E057	2.58	2.29	1.10	1.92	0.05	0.00	0.40	0.43	1.42	0.61	1.11	0.99
		CU1842 x E554	2.84	1.79	1.72	1.59	0.08	0.00	0.60	0.28	1.70	0.48	1.46	0.75
